# Inflammatory Cytokines in Maternal Circulation and Placenta of Chromosomally Abnormal First Trimester Miscarriages

**DOI:** 10.1155/2012/175041

**Published:** 2011-09-29

**Authors:** Jean Calleja-Agius, Eric Jauniaux, Shanthi Muttukrishna

**Affiliations:** ^1^UCL EGA Institute for Women's Health, University College London, 86-96, Chenies Mews, London, WCIE 6HX, UK; ^2^Department of Anatomy, Faculty of Medicine and Surgery, University of Malta, Tal-Qroqq, Msida 2090, Malta; ^3^Anu Research Centre, UCC Department of Obstetrics and Gynaecology, Cork University Maternity Hospital, 5th Floor, Wilton, Cork, Ireland

## Abstract

The impact of abnormal placental karyotype on the inflammatory response within the villous tissue and peripheral circulation of women with miscarriage was evaluated. Villous (*n* = 38) and venous blood samples (*n* = 26) were obtained from women with missed miscarriage. Tissue chromosome analysis indicated 23 abnormal and 15 normal karyotypes. Concentration of tumour necrosis factor alpha (TNF*α*), TNF-R1 and TNF-R2, and interleukin (IL)-10 were measured using flowcytometric bead array in fresh villous homogenate, cultured villous extracts, culture medium, maternal whole blood, and plasma. Plasma TNF*α*/IL-10 ratios were significantly (*P* < 0.05) lower in miscarriages with abnormal karyotype. In the abnormal karyotype group, there were significantly higher levels of TNF*α* (*P* < 0.01), IL-10 (*P* < 0.01), TNF-R1 (*P* < 0.001), and TNF-R2 (*P* < 0.001) in the villous extracts and culture-conditioned medium compared to normal karyotype group. In miscarriage with abnormal karyotype, there is an exacerbated placental inflammatory response, in contrast to miscarriage of normal karyotype where maternal systemic response is increased.

## 1. Introduction

Early pregnancy loss is the most common pregnancy complication [1]. Around 60% of first trimester miscarriages are associated with a chromosomal abnormality [2–4]. Most chromosomal abnormalities are associated with primary abnormal trophoblast invasion of the uterine decidua [5–7]. As the placental development becomes increasingly dependent on fetal synthesis towards the end of the first trimester, isolated major fetal structural defects leading to early fetal demise can also lead to secondary placental dysfunction causing a miscarriage [8]. 

Inflammatory processes taking place at the feto-maternal interface are essential for normal implantation in human pregnancy [9–11]. It has been suggested that the main regulator of this inflammatory reaction could be uterine natural killer (NK) cells [10]. Proinflammatory cytokines like tumour necrosis factor alpha (TNF*α*) have been shown to inhibit trophoblast migration through the elevation of plasminogen activator inhibitor-1 in first trimester villous explant cultures, causing abnormal trophoblast invasion [12]. TNF*α* has also been shown to downregulate the endocrine function of trophoblasts, leading to pregnancy failure [13]. It has been suggested that a network might exist in which hormones together with cytokines regulate the decidual expression of HLA-G, an antigen preferentially expressed by trophoblast, thus, maintaining maternal-fetal tolerance [14].

There is evidence of a shift in T-helper 1 (TNF*α*): T-helper 2 (interleukin, IL-10) ratio in the maternal circulation and placental villous tissue in first trimester miscarriages [15–17]. However, most previous studies have used placental tissue for early pregnancy failure without knowledge of the karyotype or have focused on women presenting with recurrent miscarriage, and thus little is known about the impact of the conceptus karyotype on the placental and systemic inflammatory responses in early pregnancy failure. The aim of this study was therefore to investigate the maternal circulatory levels, villous expression, and secretion *in vitro* of TNF*α*, TNF*α* receptors, and IL-10 and to evaluate the Th1 and Th2 cytokine ratio in early pregnancy failures with and without a chromosomal abnormality.

## 2. Materials and Methods

Chorionic villous samples (*n* = 38) were obtained from women presenting with a missed miscarriage, undergoing the evacuation of retained products of conception (ERPC) at University College London Hospital (UCLH). All women were nonsmokers, with normal body mass index (BMI) ranging between 20 and 30, not on medication, and with a history of regular menstrual cycles. The date of the last menstrual period (LMP) was used to calculate the gestational age, and women with a history of recurrent miscarriage or who did not know their LMP were excluded from the study. The gestational ages at the time of ERPC ranged between 9 weeks and 0 days and 13 weeks and 6 days gestation. Ultrasound measurement of the fetal crown-rump length (CRL) was used to evaluate the time interval between fetal demise and the surgical procedure of ERPC. 

In 12 cases with normal karyotype and 14 with abnormal karyotype, maternal peripheral venous blood (10 mL) was collected by sterile venepuncture into bottles with lithium heparin at the time of ERPC. One mL of uncoagulated blood was aspirated aseptically for whole blood analysis while the rest was centrifuged within 2 hours of collection, and the plasma supernatant was stored at −80°C until assayed.

This study was approved by the joint UCL/UCLH ethics committee on the ethics of human research. Written informed consent was obtained from each participant at the Early Pregnancy Unit prior to the surgical uterine evacuation of retained products of conception (ERPC).

### 2.1. Flowcytometric Analysis of Fluorescent Antibody-Labelled Whole Blood

Initial whole blood validation experiments showed that an incubation period of 12 hours with 40 ng/mL of lipopolysaccharide (40 LPS) gave the highest increment above basal level (0 LPS) in terms of cytokine expression by the activated viable monocytes. Dual antibody labelling was carried out with specific mouse antihuman antibodies (AbD Serotec, Oxford, UK) that were conjugated to spectrally distinct fluorochromes to identify the monocyte population (CD14) positive for the cytokine/receptor of interest. The method we used has been described elsewhere [18].

### 2.2. In Vitro Cultures

Following the ERPC, the placental villi were separated from the rest of the POC and washed twice in sterile Hank's Balanced Salt Solution with 0.1% Gentamycin Solution (Sigma-Aldrich, St Louis, USA) and 1% Amphotericin B (Invitrogen, Paisley, UK) to remove any blood. A biopsy of villous tissue measuring ~1 cm^3^ was snap frozen in liquid nitrogen and stored at −80°C until homogenised to measure the cytokine/receptor content in the villous tissue on the day of evacuation (Day 0). In all cases, a villous sample was sent to a commercial cytogenetic laboratory (TDL, London, UK) within 2 hours of the ERPC, and karyotyping was carried out by standard culturing, suspension harvest and G-band analysis methodology [19].

Villous tissue obtained from the ERPC was divided into equal sections under sterile conditions under a laminar hood and weighed. Each of the villous biopsies of known weight was cultured in 24-well culture plates containing 1 mL culture medium per well, in triplicate wells. Villous explants derived from each individual woman were cultured separately for 3 days at 6% and at 20% oxygen (O_2_) levels in a moist incubator at 37°C, 5% CO_2._ The culture medium was made up of D-MEM: F-12 (1 : 1) + GlutaMAX-I (Invitrogen, Paisley, UK) with 0.1% Gentamycin Solution (Sigma-Aldrich, St Louis, USA) and 1% Amphotericin and 1% Insulin-Transferrin-Selenium A serum supplement (Invitrogen, Paisley, UK).

Villous explant culture conditioned medium and placental villi were collected after incubation for 1 day, 2 days, and 3 days from the individual wells. Cultured villous explants were homogenized for cytokine analysis. Homogenization of villous tissue was carried out as previously described by our group [20]. All cytokines/receptors concentrations in villous explants homogenates and culture-conditioned medium were normalized against weight of tissue/well as pg/gram villous tissue.

### 2.3. Cytokine and Receptor Assays

Cytokines and receptors in plasma, culture medium, and homogenised villi were measured using BD Cytometric Bead Array (CBA) Human Soluble Protein Flex Sets and BD FACSArray bioanalyzer flowcytometer following manufacturer's instructions (BD Biosciences, San Jose, California, USA). The limit of detection was 0.7 pg/mL for TNF*α*, 0.13 pg/mL for IL-10, 5.2 pg/mL for TNF-R1, and 1.4 pg/mL for TNF-R2. The intra-assay coefficient of variation was 10.2% for TNF*α*, 6.4% for IL-10, 2.6% for TNF-R1, and 7.1% for TNF-R2. The interassay coefficient of variation was 5% for TNF*α*, 11% for IL-10, 10.1% for TNF-R1, and 5.6% for TNF-R2. The results were presented in graphical and tabular formats using the FCAP Array Software (BD Biosciences, San Jose, Calif, USA).

### 2.4. Statistical Analysis

For villous cytokine expression and the time- and O_2_-dependent villous explant medium concentrations, the data were log transformed to achieve normality, which was confirmed using the Shapiro-Wilks test and Q-Q plots. Outliers were identified using Cook's distance, and those exceeding the threshold 4/*n* were removed. 

For villous *in vivo* cytokine expression, ANOVA was performed on the log-transformed data, to estimate means for each cytokine by karyotype, adjusted for gestation at sampling. Multivariate linear regressions were carried out on the log-transformed data, to estimate the means for each cytokine by karyotype, O_2_ level (6 versus 20%), and day of culture (Day 1, 2, or 3), adjusted for gestation. For cytokine/receptor levels in the plasma and monocytes, data were normalized by log transformation, and unpaired *t*-test was carried out. 

A *P*-value less than 0.05 was considered statistically significant. The statistical analysis and graphs were produced using SPSS v.17 (SPSS Inc., Chicago, Ill, USA) and GraphPad Prism v.5 (GraphPad Software inc., San Diego, Calif, USA).

## 3. Results

Karyotyping of the products of conception (POC) indicated 15 with normal karyotype and 23 abnormal karyotypes including 16 trisomies, 4 monosomy X, and 3 triploidies. There was no difference in maternal BMI, age, parity, and ethnic distribution between the group with and without a chromosomal abnormality. The gestation at the time of ERPC, interval in days between estimated fetal demise and ERPC and fetal sex ratio was similar in both groups. In the cases where maternal blood was collected, karyotyping showed 12 normal karyotypes and 14 abnormal karyotypes.

### 3.1. Maternal Plasma Concentrations

**Figure 1:**
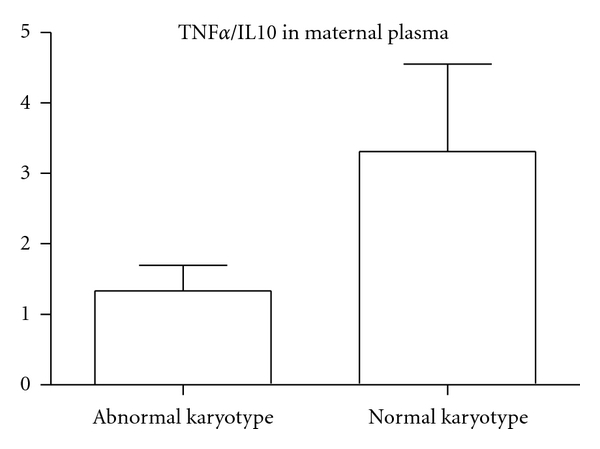
TNF*α*/IL-10 in the maternal circulation (abnormal karyotype, *n* = 14; normal karyotype, *n* = 12). There was a significantly (*P* < 0.05) lower TNF*α*/IL-10 ratio in the plasma of miscarriages of abnormal karyotype than in those with normal karyotype.

The levels of TNF*α*, TNF-R1, TNF-R2, and IL-10 were not significantly different between miscarriages with normal and abnormal karyotype. TNF*α*/IL-10 ratio in the plasma was significantly (*P* < 0.05) lower in miscarriages with an abnormal karyotype than those with normal karyotype (Figure 1).

### 3.2. Maternal Circulatory Monocyte Concentration

**Figure 2:**
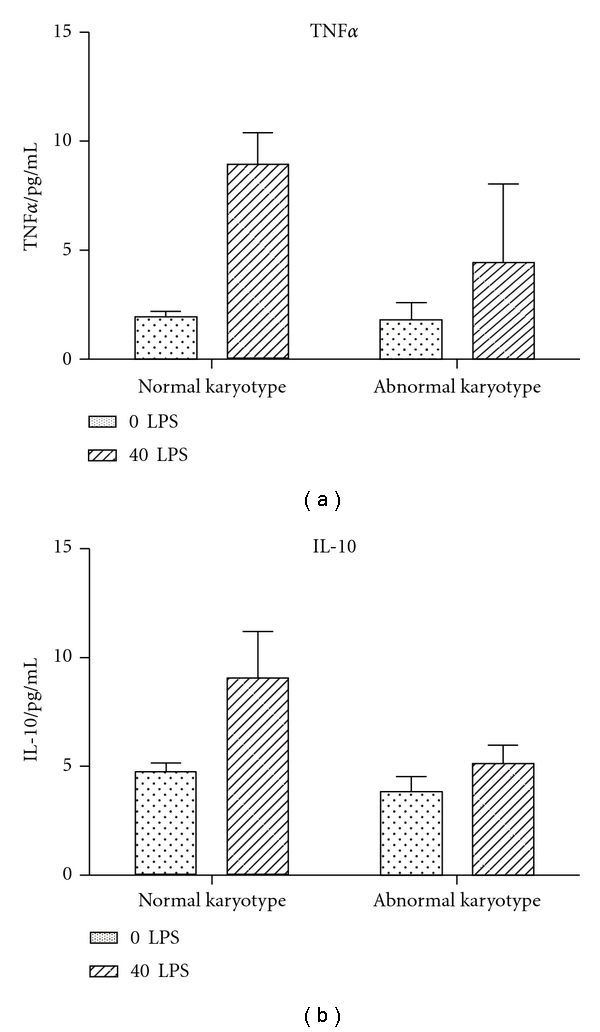
Levels of cytokines in monocytes (*n* = 12 with normal karyotype and *n* = 14 with abnormal karyotype). There was no significant difference in any of the intracellular levels of cytokines.

**Figure 3:**
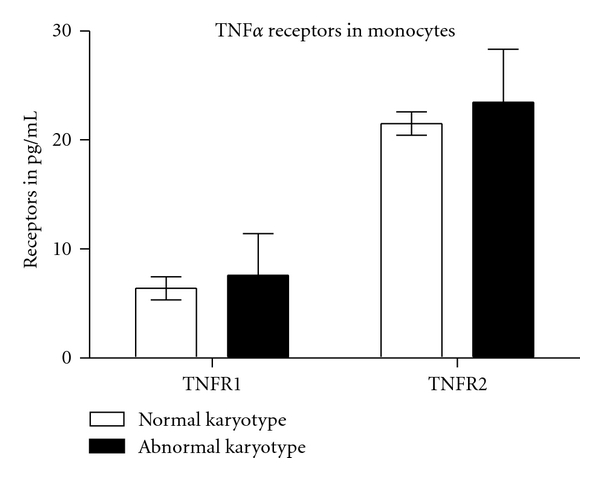
Levels of TNF-R1 and TNF-R2 in monocytes (*n* = 12 with normal karyotype and *n* = 14 with abnormal karyotype). There was no significant difference in any of the intracellular levels of receptors.

There was no significant difference between intracellular levels of TNF*α*, TNF-R1, TNF-R2, and IL-10 in the monocytes in plasma samples of both groups. There was a 3-fold higher % LPS stimulation of TNF*α* in the normal karyotype compared to the abnormal karyotype group (Figures 2 and 3).

### 3.3. Expression of Cytokine and Receptors in Villous Tissue

No significant differences were found in the cytokine and receptor levels in the snap frozen villous tissue homogenate samples between the groups presenting with normal and abnormal karyotype. These levels were similar in the different gestational subgroups, and across gestation (Table 1).

### 3.4. Villous Tissue In Vitro Secretion and Content of Cytokines and Receptors

The ANOVA test indicated that gestation has no significant impact on the relationship between cytokine/receptor levels and karyotype in culture conditions at both O_2_ concentrations.

Time in culture (1, 2, or 3 days) and O_2_ concentration (6 versus 20%) did not affect the mean cytokine/receptor levels or TNF*α*/IL-10 ratio in villous homogenates or culture-conditioned medium adjusted for gestation (Table 2).

#### 3.4.1. Villous Tissue Homogenates

Significantly higher levels of TNF*α* (*P* < 0.01), IL-10 (*P* < 0.001), and TNF-R2 (*P* < 0.001 were found in cultured villous extracts in the group with an abnormal karyotype compared to normal karyotype group. There was no significant difference in TNF*α*/IL-10 (Table 2).

#### 3.4.2. Culture Medium of Villous Tissue Samples

Significantly higher levels of TNF*α* (*P* = 0.001), IL-10 (*P* < 0.01), and TNF-R1 (*P* < 0.001) were found in the culture-conditioned medium of abnormal karyotype group compared to the normal karyotype group. There was no significant difference in TNF*α*/IL-10 (Table 2) between the groups.

## 4. Discussion

The results of our study indicate that, in sporadic miscarriages with a normal karyotype, there is an increased maternal systemic inflammatory response with an imbalance in the Th-1/Th-2 ratio in the maternal circulation. By contrast, in villous samples from the same groups, we found an increased secretion of inflammatory cytokine TNF*α* and its soluble receptors, TNF-R1 and TNF-R2, and the anti-inflammatory cytokine IL-10 in cases with an abnormal karyotype, compared to those presenting with a normal karyotype. 

We have previously shown that in early pregnancy failure, the excessive entry of oxygenated maternal blood into the intervillous space has a direct mechanical effect on the villous tissue which becomes enmeshed inside large intervillous blood thrombi and an indirect O_2_-mediated trophoblastic damage with increased apoptosis [21–23]. This phenomenon occurs at some point in miscarriages independently of the aetiology and in particular of the conceptus karyotype [24]. As all our villous tissue samples were obtained from missed miscarriage, the abnormal influx of maternal oxygenated blood would have had a similar impact on the villous tissue of both the cases presenting with and those presenting without a chromosomal karyotype at the time of the clinical diagnosis. This can explain why O_2 _and the number of days in culture had no impact on cytokine and receptor levels in our explant cultures experiments in both groups. The absence of significant difference in cytokine levels in the snap frozen samples (on day of collection) suggests that the difference seen in culture reflects functional changes. 

In the present study, we also found that in early pregnancy (i.e., before 14 weeks), the amount of TNF*α* and IL-10 secreted *in vitro *by villous tissue from sporadic miscarriage was lower in the group with a normal karyotype. These data indicate that the karyotype of the conceptus has a direct impact on the secretion of cytokines by the villous tissue. An abnormal karyotype leads to an elevated local inflammatory response, confirmed by the significant rise in TNF*α*. This was accompanied by a rise in the anti-inflammatory cytokine IL-10 and the neutralizing soluble TNF-R1 and TNF-R2. An upregulation of the cytokine expression by the villous tissue has been reported in many cases of unexplained spontaneous miscarriages associated with a severe congenital infection presenting with maternal systemic symptoms [25]. In early miscarriages, there is massive destruction of maternal immunoglobulins in embryonic monocytes, with acute villitis in the placental barrier [26]. Some authors have therefore suggested that the upregulation of cytokines is deleterious to the developing placenta and fetus, probably as a consequence of vasoconstriction and/or direct cellular damage [27, 28]. Chromosomally abnormal spontaneous miscarriages may occur because of different mechanisms than chromosomally normal spontaneous miscarriages. Chromosome aberrations cause changes in placental morphology and function, including size, shape, and vascularity and may affect rates of apoptosis of the stromal cells and cell proliferation in blood vessels during differentiation of chorionic villi [29]. 

In the maternal plasma, we observed inflammatory changes in the opposite direction than in villous tissue. The group presenting with a normal karyotype had a shift towards a Th-1 cytokine immune response in the circulation, reflected by a higher TNF*α*/IL-10 cytokine ratio than miscarriages with abnormal karyotype. Monocytes, in the normal karyotype group released a higher level of TNF*α* upon stimulation with LPS. This suggests that in the case of miscarriage of a karyotypically normal fetus, the maternal systemic inflammatory response is more sensitized. It is possible that in the case of karyotypically normal miscarriage, there is a systemic maternal immune response that causes the rejection of the fetus. In contrast, in cases with an abnormal karyotype, implantation would have already failed following the local excessive inflammatory reaction at the materno-fetal placental interface. 

In recurrent miscarriage, the circulating cytokine levels and the decidual cytokine profile are different from those found in normal first trimester controls [30–33]. In particular, during periconception in recurrent miscarriage, higher circulatory levels of NK cells [34] and higher serum levels of macrophage migration inhibition factor [35] have been shown to be predictive of miscarriage of a conceptus with normal karyotype. In addition, women with recurrent miscarriages who miscarry a pregnancy with normal karyotype have decreased serum levels of TNF*α* [36] as early as 6-7 weeks of gestation. In our study, we have only looked at spontaneous sporadic miscarriages; however, miscarriages of normal karyotype are more likely to recur due to a possible underlying maternal immune problem [37]. 

The biological functions TNF*α* depends on its binding to two known receptors, TNF-R1 and TNF-R2 [38]. The soluble forms of these membrane receptors bind to TNF*α* with high affinity and can neutralize TNF*α* function [39, 40]. Yu et al., using flowcytometric and immunohistochemical measurement of TNF-R1 in the decidua, and detection of serum levels of soluble TNF-R1 by enzyme-linked immunoassays, found an association between the over-expression of TNF-R1 and early pregnancy failure [41]. In our study, villous secretion of soluble TNF-R1 in the culture medium and soluble TNF-R2 content in villous homogenate was significantly higher in cases presenting with abnormal karyotype. 

Our data illustrate that the mechanisms leading to a miscarriage may depend on the karyotype of the conceptus. We suggest that there is a local functional disturbance in the karyotypically abnormal placental tissue while, in the case of a normal karyotype miscarriage, rejection occurs due to a maternal systemic inflammation.

## Figures and Tables

**Table 1:** *In vivo* cytokine and receptors, measured in snap frozen villous tissue collected on the day of ERPC—comparison done using 2-way ANOVA, using the variables of karyotype (normal versus abnormal) and gestation (weeks). There was no significant difference between gestation and karyotype. Estimated marginal means for each marker by karyotype, adjusted for gestation, are presented here.

Cytokine/receptor	Normal karyotype pg/g (95% CI)	Abnormal karyotype pg/g (95% CI)	Normal versus abnormal karyotype *P* value
TNF*α*	8.2 (5.3, 12.6)	7.2 (4.2, 12.2)	0.92
IL-10	2.2 (1.3, 3.8)	2.9 (1.6, 5.2)	0.79
TNF*α*/IL-10	4.5 (2.2, 9.0)	2.3 (1.3, 4.2)	0.62
TNF-R1	2499.1 (1488.8, 4195.3)	2753.26 (1927.21, 3933.4)	0.56
TNF-R2	12440.9 (6876.6, 22507.7)	12157.9 (8716.9, 16957.2)	0.68

**Table:** (a) Villous explant cultures at 6% Oxygen

Marker (pg/mL/g)	Normal karyotype						Abnormal karyotype					
	Day of culture						Day					
	1		2		3		1		2		3	
	Mean	(95% CI)	Mean	(95% CI)	Mean	(95% CI)	Mean	(95% CI)	Mean	(95% CI)	Mean	(95% CI)
TNF*α* Homo	7.0	(4.0, 12.4)	20.6	(5.3, 79.9)	6.0	(0.8, 44.9)	25.4	(14.1, 45.8)	26.9	(12.2, 59.2)	17.3	(6.7, 44.6)
IL-10 Homo	0.3	(0.2, 0.4)	0.8	(0.4, 1.9)	0.4	(0.2, 0.9)	1.0	(0.6, 1.7)	0.9	(0.4, 2.3)	1.3	(0.6, 2.9)
TNF-R2 Homo	995.9	(469.1, 2114.3)	2334.6	(740.5, 7360.2)	841.6	(274.1, 2584.4)	3159.2	(1804.8, 5530.2)	4243.4	(2901.4, 6206.2)	4279.2	(2045.4, 8952.8)
TNF-R1 Homo	314.0	(174.4, 565.5)	517.5	(252.2, 1061.9)	246.7	(161.4, 377.2)	330.3	(176.9, 616.7)	600.3	(311.3, 1157.6)	278.4	(106.7, 726.2)
TNF*α*/IL10 Homo	24.7	(14.2, 42.9)	25.5	(4.9, 131.3)	14.7	(4.1, 52.2)	22.0	(12.7, 37.9)	20.0	(10.0, 40.0)	13.4	(7.6, 23.8)
TNF*α* CM	238.6	(87.3, 651.9)	40.4	(20.4, 80.0)	53.6	(3.3, 863.7)	273.1	(156.1, 477.9)	213.2	(125.5, 362.2)	294.2	(179.1, 483.4)
IL-10 CM	134.1	(11.0, 1628.1)	2.1	(0.1, 41.6)	3.6	(0.1, 929.5)	67.1	(11.1, 404.4)	83.5	(6.2, 1116.6)	37.0	(2.5, 552.4)
TNF-R2 CM	1371.3	(504.9, 3724.8)	2276.1	(827.3, 6262.5)	2712.2	(1972.4, 3729.5)	1109.1	(364.4, 3375.9)	5120.8	(2835.5, 9248.1)	6735.4	(3554.1, 12764.3)
TNF-R1 CM	471.1	(202.5, 1095.9)	430.0	(216.5, 854.0)	472.2	(389.9, 571.8)	645.0	(478.4, 869.6)	1316.6	(882.2, 1964.9)	1186.4	(612.7, 2297.2)
TNF*α*/IL10 CM	1.8	(0.3, 9.1)	19.2	(1.8, 200.9)	15.0	(0.7, 335.4)	3.6	(0.9, 14.2)	2.9	(0.3, 24.6)	8.0	(0.7, 86.6)

**Table:** (b) Villous explant cultures at 20% oxygen

Marker (pg/mL/g)	Normal karyotype						Abnormal karyotype					
	Day of culture						Day of culture					
	1		2		3		1		2		3	
	Mean	(95% CI)	Mean	(95% CI)	Mean	(95% CI)	Mean	(95% CI)	Mean	(95% CI)	Mean	(95% CI)

TNF*α* Homo	18.9	(7.2, 50.0)	22.0	(5.0, 95.5)	2.5	(0.7, 8.7)	20.3	(10.1, 40.9)	32.6	(18.2, 58.2)	27.9	(13.4, 57.8)
IL-10 Homo	1.0	(0.4, 2.7)	0.3	(0.2, 0.7)	0.2	(0.1, 0.6)	1.9	(0.9, 3.8)	2.2	(1.0, 5.0)	1.1	(0.5, 2.4)
TNF-R2 Homo	3121.9	(1859.5, 5241.1)	1939.0	(562.8, 6680.3)	1107.7	(202.1, 6070.6)	3713.7	(2408.8, 5725.5)	4967.9	(2460.7, 10029.7)	3789.8	(2045.8, 7020.4)
TNF-R1 Homo	463.1	(218.9, 980.0)	430.6	(179.1, 1035.4)	370.6	(222.5, 617.3)	551.3	(318.5, 954.2)	645.2	(347.8, 1197.2)	738.0	(393.5, 1384.3)
TNF*α* CM	334.9	(164.4, 681.9)	44.1	(12.5, 174.8)	45.8	(5.1, 411.8)	241.8	(118.3, 494.4)	332.1	(165.8, 665.2)	218.5	(54.6, 874.2)
IL-10 CM	304.3	(43.6, 2124.4)	0.4	(0.1, 2.2)	6.5	(0.1, 846.3)	686.6	(208.8, 2257.4)	180.6	(13.7, 2376.3)	55.0	(3.7, 819.0)
TNF-R2 CM	2359.1	(1379.8, 4033.3)	2312.6	(840.1, 6365.5)	2850.6	(2717.6, 2990.2)	2681.1	(1837.1, 3912.9)	3683.9	(2561.3, 5298.4)	4874.7	(3758.0, 6323.1)
TNF-R1 CM	531.1	(338.7, 832.9)	554.5	(499.2, 615.9)	638.7	(517.2, 788.9)	661.6	(513.1, 853.1)	1177.5	(780.4, 1776.5)	1208.5	(883.4, 1653.2)
TNF*α*/IL10 Homo	18.8	(10.4, 34.1)	66.5	(24.3, 181.9)	11.0	(5.5, 22.3)	13.9	(9.4, 20.5)	14.8	(8.1, 27.2)	15.3	(7.4, 31.9)
TNF*α*/IL10 CM	1.9	(0.4, 8.6)	105.2	(13.1, 844.8)	4.0	(0.1, 365.6)	0.8	(0.3, 2.5)	1.8	(0.2, 13.6)	4.0	(0.8, 20.2)
